# Artificial Intelligence-Enhanced Electrocardiography for the Detection of Hypertrophic Cardiomyopathy in Athletes: A Systematic Review of Diagnostic Accuracy and External Validity

**DOI:** 10.7759/cureus.117692

**Published:** 2026-10-09

**Authors:** Leonardo De Medeiros Quirino, Katia X Duarte, Samia G Pereira, Giulia D Leff, Bruna C Rafael, Maria Beatriz A Pinto, Rodrigo Lopes, Cristiane S Simões, Jonas W Soares, Guilherme L Kieling

**Affiliations:** 1 Medicine, Federal University of Uberlandia, Uberlandia, BRA; 2 Medicine, Universidade Federal de Juiz de Fora (UFJF), Juiz de Fora, BRA; 3 Medicine, Universidade Ceuma, Sao Luis, BRA; 4 Medicine, Universidade Feevale, Novo Hamburgo, BRA; 5 Medicine, Faculdade Atenas de Paracatu, Paracatu, BRA; 6 Medicine, Centro Universitário Maurício de Nassau, Recife, BRA; 7 Radiology, Hospital Tacchini, Bento Gonçalves, BRA; 8 Medicine, Escola Bahiana de Medicina e Saúde Pública, Salvador, BRA; 9 Medicine, Universidade Federal do Maranhão, Sao Luis, BRA; 10 Medicine, Universidade do Sul de Santa Catarina, Tubarão, BRA

**Keywords:** artificial intelligence, athletes, diagnostic accuracy, electrocardiography, hypertrophic cardiomyopathy, sports cardiology

## Abstract

Hypertrophic cardiomyopathy (HCM) is a clinically important inherited myocardial disorder in sports cardiology because affected athletes may remain asymptomatic while being at risk of ventricular arrhythmias and sudden cardiac events. Cardiovascular screening, therefore, requires the identification of occult HCM while distinguishing pathological disease from physiological adaptations to training.

This systematic review evaluated the diagnostic accuracy and external validity of artificial intelligence-enhanced electrocardiography (AI-ECG) for detecting HCM in athletes and sports-screening populations. PubMed, Embase, CENTRAL, Scopus, Web of Science Science Citation Index Expanded, Europe PMC, IEEE Xplore, ClinicalTrials.gov, and WHO ICTRP were searched from inception through June 2026, supplemented by reference lists and citation searching. The original June 2026 records formed the authoritative review corpus; no later search update was incorporated. Eligible studies evaluated AI or machine learning applied to ECG for the detection of current HCM in athletes against an imaging-supported reference standard. Risk of bias was assessed using QUADAS-2.

The searches identified 1,024 database and registry records. After the removal of 321 duplicate occurrences and the addition of 18 records identified through supplementary methods, 721 records were screened, and 58 reports underwent full-text assessment. Fifty-six reports were excluded, one directly relevant study was ongoing without available results, and one published report was included in the qualitative synthesis. The included study evaluated 845 collegiate or professional athletes, of whom 99 underwent echocardiography, and one was identified as having HCM.

AI-ECG-derived HCM probability was not significantly associated with echocardiographically verified HCM status (r = 0.173, p = 0.086). No prespecified HCM positivity threshold, HCM-specific sensitivity, specificity, area under the receiver operating characteristic curve, or participant-level 2 × 2 diagnostic data were reported, and no study met the prespecified criteria for quantitative synthesis.

Athlete-specific evidence, therefore, remains insufficient to establish the diagnostic accuracy of AI-ECG for HCM or to support its use as a stand-alone rule-in or rule-out screening test. Prospective, participant-independent, imaging-verified external validation in representative athlete cohorts, particularly studies designed to distinguish pathological HCM from physiological athlete’s heart, is required before AI-ECG can be considered a validated component of pre-participation cardiovascular screening.

## Introduction and background

Hypertrophic cardiomyopathy (HCM) is a clinically important myocardial disorder associated with ventricular arrhythmias and sudden cardiac events [1]. Cardiovascular assessment of athletes aims to identify disease while distinguishing pathological findings from physiological adaptations to training. The resting 12-lead electrocardiography (ECG) supports this assessment, but training-related electrical remodeling can overlap with findings suggestive of disease. Athlete-specific interpretation criteria help reduce false-positive findings and unnecessary further investigation [2,3]. In a study involving 200 patients with HCM and 563 athletes, the 2017 International criteria increased specificity from 86.9% to 95.9% compared with the 2010 European Society of Cardiology criteria (p < 0.001), while sensitivity changed from 95.5% to 93% [4]. This illustrates the importance of accounting for physiological adaptation when interpreting athletes’ ECGs.

Artificial intelligence-enhanced electrocardiography (AI-ECG) may complement conventional interpretation by identifying complex combinations of ECG features and supporting standardized assessment and triage for imaging. Potential benefits include recognizing subtle disease patterns and reducing unnecessary referrals while preserving sensitivity. However, superiority over conventional athlete-specific ECG criteria has not been established by the eligible evidence directly assessed in this review. External validation is defined as testing a previously developed model in a separate population or setting, using participants who were not involved in model development or tuning.

AI-ECG has demonstrated substantial discriminatory performance for HCM in predominantly clinical populations. International external validation has reported an area under the receiver operating characteristic curve (AUC) of 0.922 with sensitivity of 82.8% and specificity of 87.7%; a retrospective HCM-center study reported an AUC of 0.91, sensitivity of 67%, and specificity of 95%; and a cardiac magnetic resonance imaging (CMR)-grounded clinical cohort reported an AUC of 0.946, sensitivity of 58%, and specificity of 100% at a prespecified threshold. However, these cohorts are not representative of athlete screening and do not establish transportability to a low-prevalence population with physiological cardiac remodeling [5-7].

We conducted a systematic review to evaluate the diagnostic accuracy and external validity of AI- and machine-learning (ML)-enhanced ECG for detecting current HCM in athletes and sports-screening populations, with particular attention to differentiation from physiological athletes’ hearts. We assessed the risk of bias and applicability and planned quantitative synthesis only when the prespecified diagnostic-accuracy requirements were met.

## Review

Methods

Protocol and Reporting

This systematic review was prospectively registered in the International Prospective Register of Systematic Reviews (PROSPERO; CRD420261423926) and was designed as a diagnostic test accuracy (DTA) review of AI-ECG for the detection of current HCM in athletes and athlete-screening populations. Reporting follows PRISMA-DTA, supplemented by PRISMA 2020 and PRISMA-S [8-10].

Review Question and PICO Framework

The review question was: “In athletes and explicitly defined athlete-screening populations, how accurately does AI-enhanced electrocardiography identify current HCM against an imaging-supported clinical reference standard, and what evidence supports its external validity?” A PICO framework adapted for DTA defined the population, index test, reference standard, and outcomes:

Population (P): Competitive, recreational, collegiate, professional, elite, or master athletes, and explicitly defined sports-cardiology or pre-participation cardiovascular screening populations. Mixed or broader clinical cohorts required separately extractable athlete-specific findings or a distinct external-validation cohort of athletes.

Index test (I): AI or ML methods applied to ECG signals to detect current HCM.

Comparator/reference standard (C): ECG using established HCM diagnostic criteria, CMR when indicated or when ECG was inconclusive, or an imaging-supported clinical diagnosis with expert adjudication. Genetic testing was considered complementary to phenotypic assessment and was not accepted as the sole reference standard.

Outcomes (O): Sensitivity and specificity as co-primary outcomes; additional outcomes included AUC, predictive values, likelihood ratios, diagnostic odds ratio, calibration, threshold-specific diagnostic data, external-validation performance, and differentiation of HCM from physiological athlete’s heart.

For this diagnostic review, the comparator was the clinical reference standard used to establish HCM status. A head-to-head comparison with conventional ECG interpretation was not a universal eligibility requirement.

Eligibility criteria

The registered eligibility criteria did not specify a restriction to a particular sport or sporting discipline, or a minimum or maximum participant age. Population eligibility was established from the study’s description of athlete status or its explicitly defined sports-screening recruitment setting. ECG features suggestive of physiological training adaptation were not used to classify participants as athletes. In mixed or broader clinical cohorts, only separately extractable athlete-specific findings or findings from a distinct athlete external-validation cohort were eligible for the direct synthesis; general clinical results were not treated as athlete-specific evidence.

Inclusion Criteria

1. Human studies evaluating athletes or explicitly defined athlete-screening populations, including mixed or broader clinical cohorts with separately extractable athlete-specific findings or a distinct external-validation cohort of athletes.

2. An AI or ML method applied to ECG signals to identify current HCM.

3. An identifiable HCM reference assessment based on ECG, CMR, or an imaging-supported clinical diagnosis with expert adjudication.

4. Prospective or retrospective diagnostic-accuracy, model-development, internal-validation, or external-validation studies, including cohort, case-control, cross-sectional, and other relevant observational designs.

5. Conference abstracts could contribute qualitative evidence when the population, index test, HCM target condition, and reported reference assessment were compatible with the review. Quantitative synthesis additionally required valid participant-level, threshold-specific diagnostic data.

Exclusion Criteria

1. General clinical or community populations without separately extractable athlete-specific findings or a distinct athlete external-validation cohort.

2. Studies without AI or ML applied to ECG signals.

3. Studies limited to left ventricular hypertrophy (LVH), ventricular mass or thickness, geometry, remodeling, physiological athlete’s heart, or other cardiomyopathies without separable HCM-specific findings.

4. Models evaluating only prognosis or future disease or event risk without classification of current HCM, and treatment or remodeling studies without relevant current-HCM diagnostic evaluation.

5. Studies lacking an acceptable imaging-supported HCM reference assessment, including studies using ECG alone, another algorithm’s prediction, or genetic testing without phenotypic evidence as the sole basis for HCM classification.

6. Reviews, editorials, opinion articles, and other reports without original relevant data; non-human, in vitro, simulation-only, and technical dataset studies without clinically defined human participants.

Participant-independent external validation was not a universal eligibility requirement. Participant overlap and ECG-vector-level splitting were considered in risk-of-bias and applicability appraisal rather than used as standalone exclusion criteria. Full-text exclusions were assigned hierarchically so that each report contributed one principal reason to PRISMA accounting. Ongoing studies without available results were described separately and contributed no diagnostic-accuracy data.

Index test, target condition, and reference standard

AI-ECG was the index test, and current HCM was the primary target condition. Eligible ECG modalities included 12-lead, single-lead, ambulatory or Holter, wearable, handheld, and smartwatch-derived ECG signals. The reference assessment used to establish HCM status was distinct from the AI-generated probability or ECG positivity threshold. Accepted reference standards were ECG using established diagnostic criteria, CMR when indicated or when ECG was inconclusive, or an imaging-supported clinical diagnosis with expert adjudication. Genetic findings were considered complementary information when linked to phenotypic assessment.

The review relied on the diagnoses and reference assessments reported by the original investigators; it did not independently diagnose participants from ECG findings. Reference-standard definitions, imaging criteria, verification fractions, and diagnostic thresholds were recorded as reported, and missing information was not inferred. Identification of an imaging-based reference assessment could support qualitative inclusion, while incomplete verification or failure to report precise HCM criteria was considered in risk-of-bias and applicability assessments. An undocumented hospital diagnosis without evidence of imaging support was insufficient. Absence of valid participant-level, threshold-specific diagnostic data precluded quantitative synthesis.

Information sources and search strategy

Searches were conducted from database inception through June 2026 in PubMed (June 22, 2026), Embase (June 19, 2026), CENTRAL (June 22, 2026), Scopus (June 19, 2026), Web of Science Core Collection - Science Citation Index Expanded (June 19, 2026), Europe PMC (June 22, 2026), IEEE Xplore (June 22, 2026), ClinicalTrials.gov (June 22, 2026), and WHO ICTRP (June 22, 2026). No date or language restrictions were applied. Search concepts covered ECG, AI and ML, HCM and related hypertrophic phenotypes, and athletes or sports screening. Source-specific search dates and original retrieval counts are reported in Appendix E. Available search documentation, strategy provenance, the complete recovered CENTRAL sequence, and source-specific correspondence checks are reported in Appendix F.

The original June 2026 search exports defined the formal review corpus and PRISMA accounting. Retrieval in some sources was broader than the four-concept operational search plan. Where the exact historical syntax could not be fully recovered, the reported strategies are explicitly identified as reconstructed. Source-specific reproducibility audits on August 17, 2026, compared the PubMed and CENTRAL rerun records with their original June archives; their methods, findings, and limitations are reported in Appendix F. These audits were not incorporated as a formal all-source search update and did not alter the original June PRISMA counts.

Supplementary identification, deduplication, and study selection

Backward reference-list checking and citation searching were performed in addition to database and registry searches. Eighteen additional records were identified through these supplementary methods and added to the screening set. Database and registry exports were consolidated and deduplicated using bibliographic identifiers and normalized-title matching, while distinct reports, conference abstracts, companion publications, and trial registrations were retained when they represented separate reports of the same study.

Screening was distributed among a team of nine reviewers in Rayyan. Each record at title- and abstract-level screening and each report at full-text assessment was evaluated by at least two reviewers, with decisions entered individually. The exported audit trail contained at least two decisions for every screened record and for each row linked to the 58-report full-text set. Conflicts at either stage were resolved by a designated adjudicator. Reports not meeting eligibility criteria were assigned a single hierarchical primary exclusion reason for PRISMA accounting.

Data extraction

Data were extracted by one reviewer using a prespecified diagnostic-accuracy form and independently checked against the source report by a second reviewer. The form captured study design, athlete population, ECG modality, AI/ML model characteristics, development and validation status, target-condition definition, reference standard, participant flow, verification fraction, diagnostic threshold, and participant-level true-positive (TP), false-negative (FN), false-positive (FP), and true-negative (TN) counts when available. Sensitivity, specificity, predictive values, likelihood ratios, diagnostic odds ratio, AUC, calibration, and subgroup results were extracted when reported. Differences were resolved by consensus, with a third reviewer designated for adjudication if required. Missing information was not inferred from unlinked publications.

Risk of bias and applicability

Risk of bias and applicability were assessed independently using QUADAS-2 by two reviewers; disagreements were resolved by consensus, with a third reviewer adjudicating unresolved domain judgments. Because the review evaluated AI-based diagnostic models, model-development and AI-specific issues, including participant-level data separation, model locking, threshold prespecification, and external validation, were additionally considered with reference to PROBAST and PROBAST+AI. The formal included-study QUADAS-2 assessment concerns the Fatunde report; appraisals of excluded or post-cutoff contextual studies were used only to support eligibility and interpretation [11-13].

Outcomes and synthesis

Sensitivity and specificity for current HCM were prespecified as co-primary outcomes. Additional outcomes included AUC, likelihood ratios, diagnostic odds ratio, positive and negative predictive values (NPVs), calibration, threshold-specific 2 × 2 data, external-validation performance, false-positive and false-negative burden, differentiation of HCM from physiological athlete's heart, and subgroup performance.

Studies were synthesized narratively according to athlete population, ECG modality, AI model type, target-condition definition, reference standard, and validation design. Diagnostic-accuracy meta-analysis was prespecified only when at least two clinically comparable studies provided valid participant-level, threshold-specific 2 × 2 data. If appropriate, sensitivity and specificity were to be jointly synthesized using a bivariate random-effects model, with a hierarchical summary receiver operating characteristic approach considered when thresholds varied meaningfully. Model-development, internal-validation, and external-validation evidence were not to be pooled indiscriminately, and correlations between model probabilities and disease status were not treated as substitutes for sensitivity or specificity. Because the quantitative-synthesis criteria were not met, no meta-analysis was performed [14,15].

Reporting bias and certainty

Where multiple sources were available for the same study, registrations, protocols, conference abstracts, preprints, and full publications were compared to identify potentially selective reporting of thresholds or performance metrics. A GRADE-informed diagnostic-accuracy certainty assessment was prespecified, but formal certainty grading was not undertaken because the directly eligible evidence did not provide threshold-specific diagnostic-accuracy estimates suitable for an evidence profile.

Results

Study Selection

The database and registry searches identified 1,024 records. After the removal of 321 duplicate occurrences, 703 unique database/registry records remained. Eighteen additional records were identified through reference-list review and citation searching, yielding 721 records for title and abstract screening. Of these, 663 reports were excluded, and 58 reports underwent full-text assessment; all 58 were retrieved. Following final eligibility adjudication, 56 reports were excluded: 43 for the wrong population, seven as duplicate or companion reports, three for the wrong target condition, one for the wrong index test, one for the wrong publication type or no original diagnostic data, and one for an inadequate or unconfirmed HCM reference standard. One published report was included in the qualitative synthesis, and one directly relevant study was ongoing without available results. The study-selection process is shown in Figure 1.

**Figure 1:**
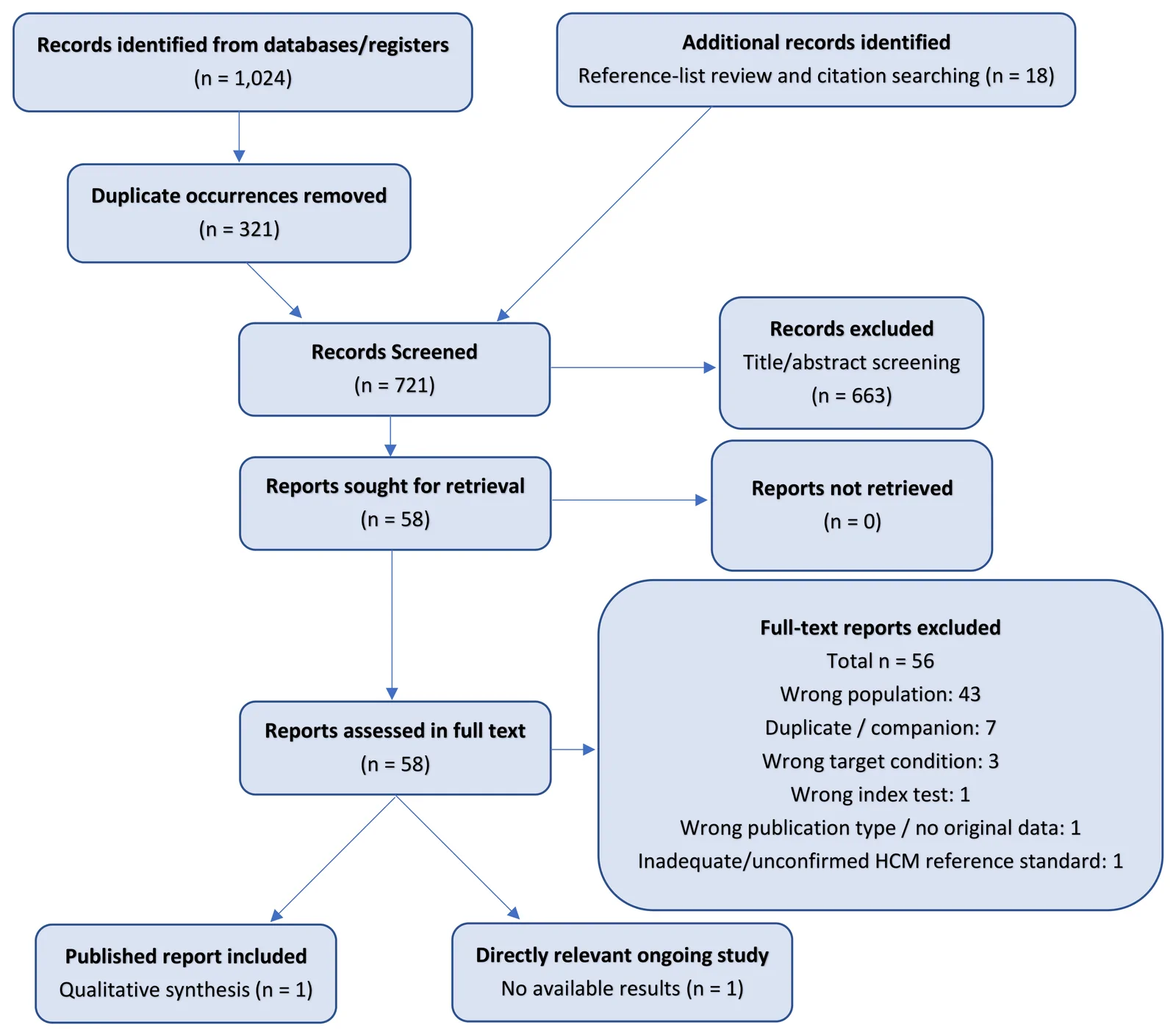
PRISMA 2020/PRISMA-DTA flow diagram. PRISMA 2020/PRISMA-DTA flow diagram of study identification, screening, eligibility assessment, and inclusion. Counts are based exclusively on the original June 2026 search corpus. DTA: diagnostic test accuracy.

Characteristics of the Included Evidence

The only published report meeting the direct eligibility criteria was a 2025 JACC conference abstract by Fatunde et al. [16] evaluating an existing AI-ECG model in a retrospective pre-participation cardiovascular screening cohort at Mayo Clinic Arizona. The study included 845 collegiate or professional athletes screened between January 2018 and September 2024. Individual sporting disciplines and the participants’ age range were not reported in the abstract. The AI-ECG generated probabilities for several phenotypes, including HCM. Follow-up ECG was available for 99 athletes, among whom one HCM case was identified. The precise echocardiographic criteria for HCM were not reported. The report did not provide a prespecified HCM positivity threshold, HCM-specific sensitivity, specificity, AUC, or participant-level 2 × 2 diagnostic data [16]. The characteristics and diagnostic evidence of the included report are summarized in Table 1. Appendix B identifies all 56 reports excluded after full-text assessment, providing bibliographic references, DOIs, or stable identifiers, along with one individual primary exclusion reason for each report.

**Table 1:** Characteristics and diagnostic evidence of the included athlete report. Study characteristics and reported results are from Fatunde et al. [16]. Eligibility and QUADAS-2 judgments were assigned by the review authors. The reported correlation is not a measure of threshold-specific diagnostic accuracy. AI: artificial intelligence; AI-ECG: artificial intelligence-enhanced electrocardiography; AUC: area under the receiver operating characteristic curve; ECG: electrocardiogram; HCM: hypertrophic cardiomyopathy; QUADAS-2: Quality Assessment of Diagnostic Accuracy Studies 2; r: correlation coefficient; p: p-value.

Characteristic	Fatunde et al. (2025) [16]
Publication and identifier	Fatunde et al. (2025) [16]; conference abstract. J Am Coll Cardiol. 2025;85(12 Suppl):2501. DOI: 10.1016/S0735-1097(25)02985-7.
Design and setting	Retrospective pre-participation cardiovascular screening cohort at Mayo Clinic Arizona, January 2018 to September 2024.
Population	845 collegiate or professional athletes undergoing pre-participation cardiovascular screening. Individual sporting disciplines and participant age range were not reported in the abstract.
Index test	Existing AI-ECG model applied to screening ECGs, generating HCM probability outputs. Model version and locking status were not reported.
Reference standard	Follow-up ECG in 99 of 845 athletes. HCM imaging criteria, reader blinding, and ECG-to-imaging timing were not reported.
Verified HCM and remaining participants	One HCM case among 99 athletes with echocardiographic assessment. HCM status in the remaining 746 athletes was not established by the reported reference assessment.
HCM positivity threshold	Not reported.
Reported statistical result	Correlation between AI-ECG HCM probability and echocardiographically verified HCM status: r = 0.173; p = 0.086.
Diagnostic-accuracy data	No reported HCM-specific sensitivity, specificity, AUC, or threshold-specific participant-level 2 × 2 diagnostic data.
Reason for qualitative inclusion	Athlete screening population; AI applied to ECG for current HCM; echocardiographic reference assessment reported. Insufficient data for quantitative synthesis.
Review appraisal and contribution	QUADAS-2: overall risk of bias High; overall applicability concern unclear. Included in qualitative synthesis only; no diagnostic-accuracy meta-analysis.

Diagnostic Performance for HCM

Among athletes who underwent ECG, AI-ECG-derived HCM probability was not significantly associated with echocardiographically verified HCM status (r = 0.173, p = 0.086). The investigators considered the HCM analysis underpowered and emphasized the need for larger athlete cohorts. Because no HCM positivity threshold or participant-level contingency data were reported, threshold-specific diagnostic accuracy could not be calculated or reconstructed [16].

Excluded Proof-of-Concept Evidence

Adetiba et al. [17] evaluated an artificial neural network using ECG data from 40 participants assigned to four diagnostic classes. Ten participants in the HCM class were active or retired professional athletes, but comparator classes mixed athletes and non-athletes. The development dataset comprised 400 ECG vectors, and training, validation, and testing were split at the ECG-vector rather than participant level. A later generalization set used one additional ECG vector from each of the same 40 participants. Although all 10 HCM vectors in that set were classified as HCM, the study was excluded because the published report described the HCM reference standard only as a prior hospital diagnosis by a certified medical professional, without documented echocardiographic or CMR criteria or imaging-supported expert adjudication. This was the single primary exclusion reason. ECG-vector-level splitting and reuse of participants were considered additional methodological limitations rather than separate eligibility exclusions. The reported performance was therefore not used for quantitative inference [17].

Ongoing Evidence

One directly relevant prospective study (NCT06290570) was identified. The study is designed to evaluate AI-ECG for the detection of HCM and differentiation from athlete's heart using standard 12-lead ECG and consumer-facing ECG devices. No result-bearing publication or posted diagnostic-performance results were available for the current evidence synthesis; the study therefore contributed no diagnostic-accuracy data [18].

Risk of Bias and Applicability

The final consensus QUADAS-2 assessment judged Fatunde et al. [16] at high overall risk of bias and with unclear overall applicability. Patient selection was at unclear risk of bias and low applicability concern because consecutive or random enrollment and exclusions were not fully reported, although the athlete-screening population was directly relevant. The index test was at unclear risk of bias and low applicability concern because model version and locking status and a prespecified HCM positivity threshold were not reported. The reference standard was at unclear risk of bias and unclear applicability concern because ECG was acceptable in principle, but HCM criteria, reader blinding, and ECG-to-imaging timing were not reported. Flow and timing were at high risk of bias because only 99 of 845 athletes underwent the reported echocardiographic reference standard; applicability is not assessed for this domain. B.C.R. adjudicated the reference-standard, flow-and-timing, and overall consensus judgments [11,16].

Quantitative Synthesis

The prespecified diagnostic-accuracy meta-analysis was not performed because the quantitative-synthesis criteria were not met. No two eligible, clinically comparable studies provided valid participant-level, threshold-specific 2 × 2 diagnostic data. The included Fatunde report provided a correlation between AI-ECG HCM probability and echocardiographically verified HCM rather than threshold-specific diagnostic-accuracy estimates. Accordingly, pooled sensitivity, specificity, likelihood ratios, diagnostic odds ratio, bivariate random-effects estimates, and hierarchical summary receiver operating characteristic models were not calculated. The overall research landscape and the relationship between direct athlete evidence, indirect clinical HCM evidence, ongoing research, and contextual post-cutoff evidence are summarized in Figure 2.

**Figure 2:**
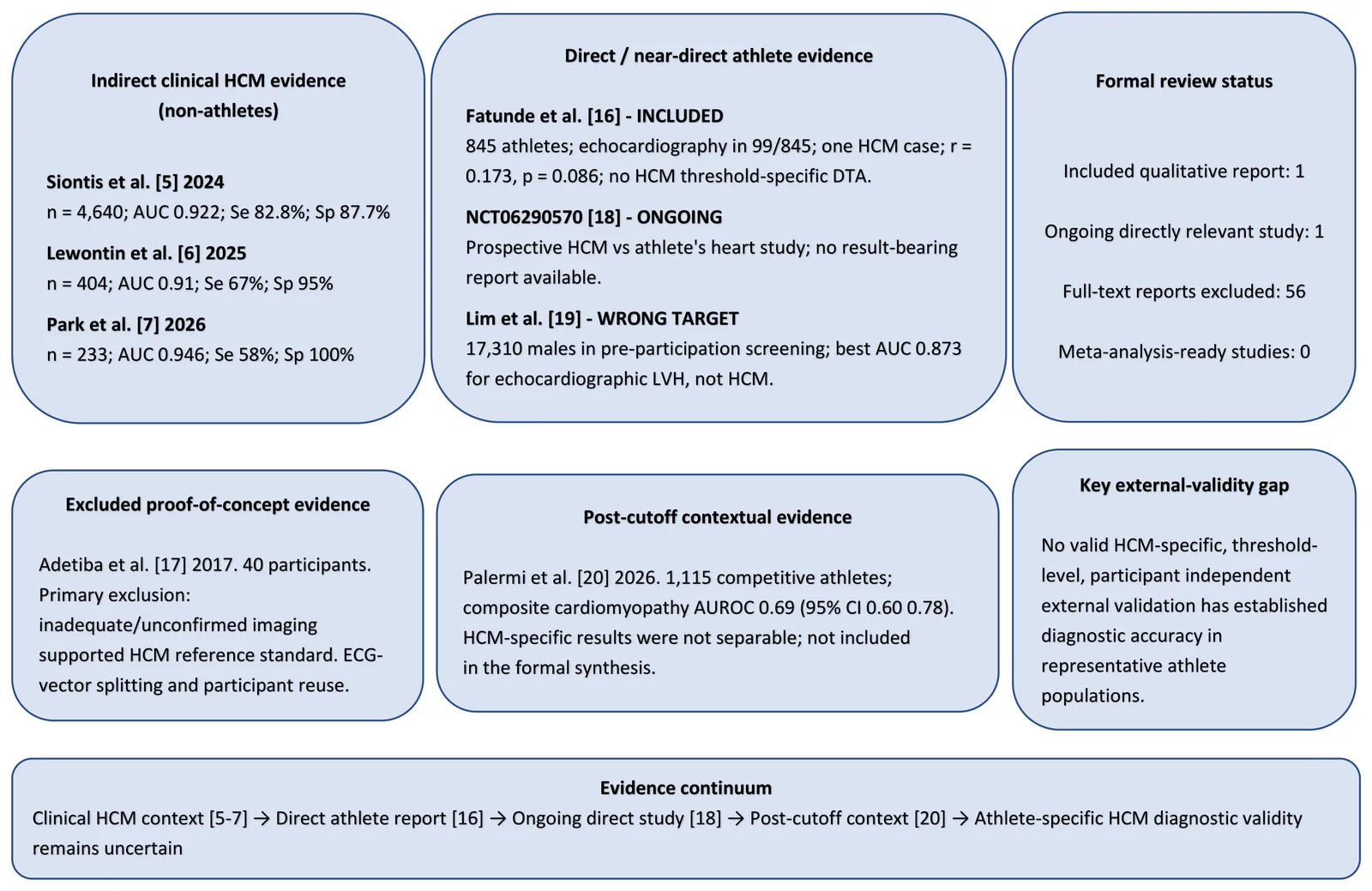
Research landscape and evidence map for AI-ECG detection of HCM in athletes. Evidence map of direct athlete evidence, indirect clinical context, full-text exclusions, ongoing research, and post-cutoff contextual evidence. Only evidence from the original June 2026 search corpus contributed to the formal systematic review; later evidence is shown for context only.

Discussion

Principal Findings

This systematic review demonstrates a substantial gap between the rapid development of AI-ECG for HCM and the evidence required to support its use in athlete screening. Despite broad searching across biomedical, engineering, and trial-registry sources, only one published report met the prespecified criteria for direct qualitative evidence in athletes, and no eligible study provided protocol-valid, threshold-specific diagnostic data suitable for meta-analysis. The central finding is therefore not that AI-ECG lacks potential for detecting HCM, but that HCM-specific diagnostic accuracy has not yet been adequately validated in representative athlete populations.

The directly eligible Fatunde report evaluated AI-ECG in 845 collegiate or professional athletes, but only 99 underwent reported follow-up ECG, and only one HCM case was identified among those verified. The publication did not report an HCM threshold, sensitivity, specificity, HCM-specific AUC, or participant-level 2 × 2 data. It therefore could not provide a reliable estimate of HCM diagnostic accuracy in athletes [16].

Contrast With the Broader Clinical HCM Literature

The paucity of athlete-specific evidence contrasts with the performance reported in clinical HCM cohorts. Siontis et al. [5] externally validated an AI-ECG algorithm across international cohorts and reported an AUC of 0.922, sensitivity of 82.8%, and specificity of 87.7% at an HCM probability threshold of 11%, carried forward from model derivation. Lewontin et al. [6] reported an AUC of 0.91, sensitivity of 67%, and specificity of 95% in a retrospective HCM-center cohort; the authors described cutoffs designed to favor specificity and positive predictive value (PPV) but did not report a numerical positivity threshold. Park et al. [7], using a CMR-grounded clinical cohort, reported an AUC of 0.946, sensitivity of 58%, and specificity of 100% at a prespecified probability threshold of 0.84.

Appendix G summarizes the HCM target or reference definitions, conventional athlete ECG criteria, and AI positivity thresholds used in the reports discussed [4-7,16]. Imaging criteria used to confirm HCM must be distinguished from the ECG operating point used to flag suspected disease. AI probability thresholds are model-specific and cannot be assumed transferable to athlete screening. The included Fatunde report did not provide an HCM positivity threshold or threshold-specific diagnostic-accuracy estimates [16]. Consequently, the directly eligible evidence cannot establish whether an athlete-specific AI threshold should differ from clinical thresholds or whether AI-ECG improves on conventional athlete-specific ECG criteria.

Why Clinical Performance May Not Transport To Athletes

Transportability is especially important in sports cardiology because athlete screening differs from referral-based HCM cohorts in disease prevalence, phenotype spectrum, physiology, and intended clinical use. Athletic training produces electrical and structural adaptations that may overlap with pathological hypertrophy, while HCM prevalence in a screening setting is far lower than in enriched case-control or specialist cohorts. These differences can produce spectrum and domain shift, changing threshold-dependent sensitivity, specificity, predictive values, calibration, referral burden, and downstream imaging use [2-4].

In a low-prevalence screening environment, modest reductions in specificity can generate substantial false-positive referrals, whereas inadequate sensitivity may provide false reassurance. Therefore, discrimination established in hospital-derived populations cannot be equated with clinical utility in athlete screening.

Physiological Hypertrophy and Target-Condition Specificity

The review identified evidence that ML can detect hypertrophic phenotypes in young screening populations. In a cohort of 17,310 males aged 16-23 years undergoing pre-participation medical screening, ML ECG models achieved an AUC of up to 0.873 for echocardiographic LVH. However, LVH was defined by left ventricular mass index and is not synonymous with HCM. The clinically relevant challenge is more demanding: distinguishing pathological HCM from physiological athlete's heart and other causes of increased wall thickness [19].

Verification and the Fatunde Study

Fatunde et al. [16] illustrates a central methodological challenge in athlete-screening validation: obtaining an adequate reference standard across the screened cohort. Only 99 of 845 athletes underwent reported follow-up ECG, creating substantial partial-verification concern, and only one HCM event was observed among verified participants. The reported association between AI-ECG HCM probability and HCM status should not be interpreted as diagnostic failure or success because correlation is not equivalent to threshold-specific diagnostic accuracy, and the analysis contained only one verified HCM event [16].

The most defensible interpretation is that the study was insufficiently powered and incompletely verified to establish athlete-specific HCM diagnostic performance. This distinction is important because a null correlation in such a sparse setting does not demonstrate absence of diagnostic signal.

Excluded Proof-of-Concept Evidence and Participant Leakage

Adetiba et al. was excluded because the published report did not document an accepted imaging-supported HCM reference standard. Its ECG-vector-level data split and reuse of participants in the later generalization set also raise concerns about potential data leakage and optimistic performance estimates. These were methodological limitations, not additional primary exclusion reasons. The excluded study was discussed qualitatively and did not contribute to the formal included-study QUADAS-2 assessment [17].

Post-Search Contextual Evidence and Domain Shift

A particularly informative study appeared after the formal June search cutoff. Palermi et al. [20] evaluated hospital-trained AI-ECG models in 1,115 competitive athletes reserved for true external validation without fine-tuning or recalibration. For the composite cardiomyopathy endpoint (HCM, dilated cardiomyopathy, or left ventricular systolic dysfunction) the AUROC decreased from 0.89 internally to 0.69 externally, an absolute decline of 0.20. At an exploratory Youden threshold, sensitivity and specificity were both 73%, with PPV 6% and NPV 98%.

These results provide contextual evidence of domain shift in real-world athlete screening. However, the external endpoint was composite cardiomyopathy, not HCM alone, and HCM-specific case counts and diagnostic-performance estimates were not separable. Palermi et al. [20] therefore does not enter the primary HCM synthesis or June PRISMA flow, but it reinforces the review's external-validity interpretation.

Clinical Implications

Current athlete-specific evidence is insufficient to support AI-ECG as a stand-alone rule-in or rule-out test for HCM in athletes. There is currently no reliable athlete-specific estimate of sensitivity or specificity on which a screening pathway could be based. If AI-ECG is considered clinically before definitive athlete-specific validation, it is more appropriately conceptualized as an adjunct to established pre-participation assessment rather than a replacement for expert ECG interpretation or definitive cardiac imaging.

Research Priorities

Future studies should prioritize prospective, multicenter external validation in representative athlete populations with sufficiently complete ECG and/or CMR verification. Data separation must occur at the participant level; model development, tuning, internal testing, and external validation populations must remain independent; the deployed model should be locked before external evaluation; and positivity thresholds should be prespecified rather than optimized retrospectively in the test cohort. Publications should report participant-level TP, FN, FP, and TN together with sensitivity, specificity, predictive values, likelihood ratios, discrimination, and calibration.

Validation cohorts should specifically include physiological athlete's heart and other conditions producing increased wall thickness rather than relying on generic healthy controls. Subgroup performance by sex, age, race or ethnicity, sport, training volume, HCM phenotype, and ECG modality should be reported. NCT06290570 is directly aligned with this evidence gap because it is designed to evaluate AI-ECG detection of HCM and distinction from athlete's heart using standard and consumer-facing ECG devices [18].

Strengths and Limitations

Strengths of this review include prospective registration, broad biomedical, engineering, and registry coverage, explicit athlete-specific eligibility, hierarchical full-text exclusion rules, supplementary citation searching, and a prespecified DTA synthesis framework that prevented inappropriate pooling. The review also maintained a strict distinction between athlete-specific diagnostic evidence and indirect clinical HCM evidence.

The principal limitation is the extreme scarcity and weakness of the directly eligible evidence. Only one published report was included, it was a conference abstract, echocardiographic verification was incomplete, only one HCM case was identified among verified athletes, and threshold-specific DTA data were unavailable. Individual sporting disciplines, participant age range, and precise HCM imaging criteria were not reported, limiting assessment of applicability across sports and age groups and interpretation of the reference standard [16]. Consequently, meta-analysis, formal heterogeneity investigation, and meaningful assessment of publication bias were not possible. A second limitation relates to search reproducibility: the archived June retrieval sets in some sources were broader than the written four-concept operational search plan. The actual archived corpus was retained as the authoritative record, and the distinction between planned architecture and executed retrieval is reported transparently rather than retrospectively altering PRISMA counts.

## Conclusions

AI-ECG has demonstrated substantial potential for HCM detection in general clinical populations, but athlete-specific diagnostic validity remains insufficiently established. Only one published report met the direct eligibility criteria, and its limited reference-standard verification and very small number of confirmed HCM cases prevented reliable estimation of sensitivity or specificity. No study provided protocol-valid data suitable for diagnostic accuracy meta-analysis. Prospective, participant-independent, imaging-verified external validation in representative athlete populations, particularly studies designed to distinguish pathological HCM from physiological athlete's heart, is required before AI-ECG can be considered a validated component of pre-participation cardiovascular screening.

## Appendices

Appendix A: Search transparency and evidence lock

The formal systematic-search corpus consisted exclusively of the original June 2026 archive. No later retrieval was incorporated as a formal search update, and no later record altered the PRISMA identification or eligibility counts. The original source files contained 1,024 raw database/register occurrences before deduplication.

Where exact executed historical syntax could not be fully recovered, this is explicitly indicated rather than retrospectively replacing the original search with a planned or reconstructed query. The original June 2026 search exports therefore remained the authoritative corpus for study identification, screening, eligibility assessment, and PRISMA accounting.

After the removal of 321 duplicate occurrences, 703 unique database/registry records remained. Eighteen additional records were identified through reference-list review and citation searching, resulting in 721 records screened. Of these, 663 were excluded during title/abstract screening, and 58 reports underwent full-text assessment. All 58 reports were retrieved. Fifty-six reports were excluded after full-text assessment, one published report was included in the qualitative synthesis, and one directly relevant study was ongoing without available results. No study met the prespecified requirements for quantitative diagnostic-accuracy synthesis.

The 56 full-text exclusions comprised 43 reports with the wrong population, seven duplicate or companion reports, three with the wrong target condition, one with the wrong index test, one with the wrong publication type/no original diagnostic data, and one with an inadequate or unconfirmed hypertrophic cardiomyopathy (HCM) reference standard.

Appendix B: Full-text exclusion criteria

Full-text exclusions were assigned hierarchically so that each excluded report contributed one principal reason to PRISMA accounting.

Wrong population (n = 43): Studies were excluded when they did not include a separable athlete or sports-screening population or athlete-specific external validation.

Duplicate or companion report (n = 7): Duplicate publications and companion reports describing the same underlying study population were consolidated and were not counted as independent studies.

Wrong target condition (n = 3): These studies evaluated related outcomes such as echocardiographic left ventricular hypertrophy (LVH) or general cardiovascular risk rather than current HCM.

Wrong index test (n = 1): One directly relevant study evaluated conventional ECG interpretation criteria rather than artificial intelligence (AI) or machine learning (ML) applied to ECG.

Wrong publication type/no original diagnostic data (n = 1): One narrative review did not provide original participant-level diagnostic-accuracy data.

Inadequate/unconfirmed HCM reference standard (n = 1): Adetiba et al. [17] described prior hospital diagnosis by a certified medical professional without documented echocardiographic or CMR HCM criteria or imaging-supported expert adjudication. The imaging-supported HCM reference standard could therefore not be confirmed. This was the single primary exclusion reason; ECG-vector splitting and participant reuse were discussed separately as methodological limitations.

**Table 2:** Reports excluded after full-text assessment. The table identifies all 56 reports excluded after full-text assessment, retaining the original inventory numbers and one primary exclusion reason per report. Records 11 (Fatunde et al. [16], included in the qualitative synthesis) and 57 (NCT06290570, ongoing) are presented separately. Palermi et al. [20] is a separate post-cutoff contextual report and is outside this exclusion inventory. AI: artificial intelligence; CMR: cardiac magnetic resonance; DOI: digital object identifier; ECG: electrocardiogram; HCM: hypertrophic cardiomyopathy; LVH: left ventricular hypertrophy; ML: machine learning.

No.	Bibliographic reference and identifier	Primary exclusion reason	Specific rationale
1	Lewontin M, Kaplan E, Bilchick KC, et al. Advanced Diagnosis of Hypertrophic Cardiomyopathy with AI-ECG and Differences Based on Ethnicity and HCM Subtype. J Clin Med. 2025 Jul 3;14(13):4718.	Wrong population	No separable athlete cohort or athlete-specific external validation; under the prespecified hierarchy this criterion is applied before target-condition or downstream technical exclusions.
2	Kwon JM, Jeon KH, Kim HM, et al. Comparing the performance of artificial intelligence and conventional diagnosis criteria for detecting left ventricular hypertrophy using electrocardiography. Europace. 2020 Mar 1;22(3):412-419. DOI: 10.1093/europace/euz324	Wrong population	No separable athlete cohort or athlete-specific external validation; under the prespecified hierarchy this criterion is applied before target-condition or downstream technical exclusions.
3	Angelaki E, Marketou ME, Barmparis GD, et al. Detection of abnormal left ventricular geometry in patients without cardiovascular disease through machine learning: An ECG-based approach. J Clin Hypertens (Greenwich). 2021 May;23(5):935-945. DOI: 10.1111/jch.14200	Wrong population	No separable athlete cohort or athlete-specific external validation; under the prespecified hierarchy this criterion is applied before target-condition or downstream technical exclusions.
4	Sparapani R, Dabbouseh NM, Gutterman D, et al. Detection of Left Ventricular Hypertrophy Using Bayesian Additive Regression Trees: The MESA. J Am Heart Assoc. 2019 Mar 5;8(5):e009959. DOI: 10.1161/jaha.118.009959	Wrong population	No separable athlete cohort or athlete-specific external validation; under the prespecified hierarchy this criterion is applied before target-condition or downstream technical exclusions.
5	Zhou Q, Luo X, Du K. Interpretable detection of left ventricular hypertrophy using commercial ECG features and machine learning: a study based on the PTB-XL+ dataset. Front Cardiovasc Med. 2026 Jun 4;13:1825829. DOI: 10.3389/fcvm.2026.1825829	Wrong population	No separable athlete cohort or athlete-specific external validation; under the prespecified hierarchy this criterion is applied before target-condition or downstream technical exclusions.
6	Naderi H, Ramírez J, van Duijvenboden S, et al. Predicting left ventricular hypertrophy from the 12-lead electrocardiogram in the UK Biobank imaging study using machine learning. Eur Heart J Digit Health. 2023 Jun 1;4(4):316-324. DOI: 10.1093/ehjdh/ztad037	Wrong population	No separable athlete cohort or athlete-specific external validation; under the prespecified hierarchy this criterion is applied before target-condition or downstream technical exclusions.
7	Suppah M, Roehl K, Lew K, et al. Assessment of Positive Cardiac Remodeling in Hypertrophic Obstructive Cardiomyopathy Using an Artificial Intelligence-Based Electrocardiographic Platform in Patients Treated With Mavacamten. Mayo Clin Proc Digit Health. 2024 Apr 10;2(2):255-257. DOI: 10.1016/j.mcpdig.2024.04.002	Wrong population	No separable athlete cohort or athlete-specific external validation; under the prespecified hierarchy this criterion is applied before target-condition or downstream technical exclusions.
8	Tang W, Cui K, Chan RH, et al. Bilateral Signal Warping for Left Ventricular Hypertrophy Diagnosis. 2025 IEEE 22nd International Symposium on Biomedical Imaging (ISBI). 2025:1-5. DOI: 10.1109/ISBI60581.2025.10980912	Wrong population	No separable athlete cohort or athlete-specific external validation; under the prespecified hierarchy this criterion is applied before target-condition or downstream technical exclusions.
9	Sparapani R, Dabbouseh N, Gutterman D, et al. Novel electrocardiographic criteria for the diagnosis of left ventricular hypertrophy derived with bayesian additive regression trees: the multi-ethnic study of atherosclerosis. Circulation. 2018;138. Conference abstract. CENTRAL: CN-01935954	Duplicate/companion report	Duplicate/companion of full-text record 4; merge with the primary report before applying clinical criteria.
10	Cleveland Clinic. AI-Enhanced ECG Analysis Shows Strong Accuracy in Detecting Hypertrophic Cardiomyopathy. Consult QD. 2 April 2025. Stable URL: Consult QD article	Duplicate/companion report	Duplicate/companion of full-text record 12; merge with the primary report before applying clinical criteria.
11	Desai MY, Jadam S, Abusafia M, et al. Real-World Artificial Intelligence-Based Electrocardiographic Analysis to Diagnose Hypertrophic Cardiomyopathy. JACC Clin Electrophysiol. 2025 Jun;11(6):1324-1333. DOI: 10.1016/j.jacep.2025.02.024	Wrong population	No separable athlete cohort or athlete-specific external validation; under the prespecified hierarchy this criterion is applied before target-condition or downstream technical exclusions.
12	Haimovich JS, Diamant N, Khurshid S, et al. Artificial intelligence-enabled classification of hypertrophic heart diseases using electrocardiograms. Cardiovasc Digit Health J. 2023 Mar 7;4(2):48-59. DOI: 10.1016/j.cvdhj.2023.03.001	Wrong population	No separable athlete cohort or athlete-specific external validation; under the prespecified hierarchy this criterion is applied before target-condition or downstream technical exclusions.
13	Huang JT, Tseng CH, Huang WM, et al. Comparison of machine learning and conventional criteria in detecting left ventricular hypertrophy and prognosis with electrocardiography. Eur Heart J Digit Health. 2025 Feb 11;6(2):252-260. DOI: 10.1093/ehjdh/ztaf003	Wrong population	No separable athlete cohort or athlete-specific external validation; under the prespecified hierarchy this criterion is applied before target-condition or downstream technical exclusions.
14	Yu R, Jia L, Pu Y, et al. EFFNet: Efficient feature fusion network for left ventricular hypertrophy identification based on 12-lead electrocardiogram signals. Heart Rhythm. 2026 Mar 4:S1547-5271(26)00204-3. Online ahead of print. DOI: 10.1016/j.hrthm.2026.02.046	Wrong population	No separable athlete cohort or athlete-specific external validation; under the prespecified hierarchy this criterion is applied before target-condition or downstream technical exclusions.
15	Liu CW, Wu FH, Hu YL, et al. Left ventricular hypertrophy detection using electrocardiographic signal. Research Square [preprint]. 2022. Version 1. DOI: 10.21203/rs.3.rs-1865396/v1	Wrong population	No separable athlete cohort or athlete-specific external validation; under the prespecified hierarchy this criterion is applied before target-condition or downstream technical exclusions.
16	Lim DY, Sng G, Ho WHH, et al. Machine learning versus classical electrocardiographic criteria for echocardiographic left ventricular hypertrophy in a pre-participation cohort. Kardiol Pol. 2021;79(6):654-661. DOI: 10.33963/kp.15955	Wrong target condition	Relevant athlete/pre-participation population and ML-ECG index test, but the reference target is echocardiographic LVH rather than current HCM.
17	Li A, Wang Y, Chen H. AI driven cardiovascular risk prediction using NLP and Large Language Models for personalized medicine in athletes. SLAS Technol. 2025 Jun;32:100286. DOI: 10.1016/j.slast.2025.100286	Wrong target condition	Athlete population/context, but the target is broad cardiovascular-risk prediction rather than diagnosis of current HCM.
18	Adetiba E, Iweanya VC, Popoola SI, et al. Automated detection of heart defects in athletes based on electrocardiography and artificial neural network. Cogent Engineering. 2017;4(1):1411220. DOI: 10.1080/23311916.2017.1411220	Inadequate/unconfirmed HCM reference standard	The report documents prior hospital diagnosis by a certified medical professional, without documented echocardiographic/CMR HCM criteria or imaging-supported expert adjudication.
19	Kosaraju E. CardiAWARE: A Novel ECG-based Deep Neural Network Algorithm for Early Detection of Cardiac Conditions. 2023 IEEE MIT Undergraduate Research Technology Conference (URTC). 2023:1-4. DOI: 10.1109/URTC60662.2023.10535038	Wrong population	No separable athlete cohort or athlete-specific external validation; under the prespecified hierarchy this criterion is applied before target-condition or downstream technical exclusions.
20	Mahin MA, Ahmad M. Diagnosis of Left Ventricular Hypertrophy from ECG Signals Based on CCS Methodology using SVM. 2021 5th International Conference on Electrical Engineering and Information Communication Technology (ICEEICT). 2021:1-6. DOI: 10.1109/ICEEICT53905.2021.9667877	Wrong population	No separable athlete cohort or athlete-specific external validation; under the prespecified hierarchy this criterion is applied before target-condition or downstream technical exclusions.
21	Sinha N, Joshi AM. Predicting the presence of Left Ventricular Hypertrophy using Este’s Criteria with Support Vector Machine. Research Square [preprint]. 2022. Version 1. DOI: 10.21203/rs.3.rs-1626968/v1	Wrong population	No separable athlete cohort or athlete-specific external validation; under the prespecified hierarchy this criterion is applied before target-condition or downstream technical exclusions.
22	Lim DY, Sng G, Ho WHH, et al. Machine learning versus classical electrocardiographic criteria for echocardiographic left ventricular hypertrophy in a pre-participation cohort. Kardiol Pol. 2021;79(6):654-661. DOI: 10.33963/kp.15955	Duplicate/companion report	Duplicate/companion of full-text record 17; merge with the primary report before applying clinical criteria.
23	Siontis KC, Wieczorek MA, Maanja M, et al. Hypertrophic cardiomyopathy detection with artificial intelligence electrocardiography in international cohorts: an external validation study. Eur Heart J Digit Health. 2024 Apr 15;5(4):416-426. DOI: 10.1093/ehjdh/ztae029	Wrong population	No separable athlete cohort or athlete-specific external validation; under the prespecified hierarchy this criterion is applied before target-condition or downstream technical exclusions.
24	Liu CM, Hsieh ME, Hu YF, et al. Artificial Intelligence-Enabled Model for Early Detection of Left Ventricular Hypertrophy and Mortality Prediction in Young to Middle-Aged Adults. Circ Cardiovasc Qual Outcomes. 2022 Aug;15(8):e008360. DOI: 10.1161/circoutcomes.121.008360	Wrong population	No separable athlete cohort or athlete-specific external validation; under the prespecified hierarchy this criterion is applied before target-condition or downstream technical exclusions.
25	Tison GH, Zhang J, Delling FN, et al. Automated and Interpretable Patient ECG Profiles for Disease Detection, Tracking, and Discovery. Circ Cardiovasc Qual Outcomes. 2019 Sep;12(9):e005289. DOI: 10.1161/circoutcomes.118.005289	Wrong population	No separable athlete cohort or athlete-specific external validation; under the prespecified hierarchy this criterion is applied before target-condition or downstream technical exclusions.
26	Yang ZY, Hsing SC, Tsai DJ, et al. Deep learning-enabled ECG system for detecting left ventricular hypertrophy and predicting cardiovascular prognoses. BioData Min. 2026 Mar 4;19(1):25. DOI: 10.1186/s13040-026-00536-2	Wrong population	No separable athlete cohort or athlete-specific external validation; under the prespecified hierarchy this criterion is applied before target-condition or downstream technical exclusions.
27	Dhingra LS, Aminorroaya A, Sangha V, et al. Ensemble Deep Learning Algorithm for Structural Heart Disease Screening Using Electrocardiographic Images: PRESENT SHD. J Am Coll Cardiol. 2025 Apr 1;85(12):1302-1313. DOI: 10.1016/j.jacc.2025.01.030	Wrong population	No separable athlete cohort or athlete-specific external validation; under the prespecified hierarchy this criterion is applied before target-condition or downstream technical exclusions.
28	Dwivedi T, Xue J, Treiman D, et al. Machine learning models of 6-lead ECGs for the interpretation of left ventricular hypertrophy (LVH). J Electrocardiol. 2023 Mar-Apr;77:62-67. DOI: 10.1016/j.jelectrocard.2022.12.001	Wrong population	No separable athlete cohort or athlete-specific external validation; under the prespecified hierarchy this criterion is applied before target-condition or downstream technical exclusions.
29	Altintepe A, Rustemli A, Vazifeh AR, et al. An Open-Source Analysis of Cardiomyopathy Using Machine Learning and Electrocardiograms. Diagnostics (Basel). 2026 Feb 28;16(5):719. DOI: 10.3390/diagnostics16050719	Wrong population	No separable athlete cohort or athlete-specific external validation; under the prespecified hierarchy this criterion is applied before target-condition or downstream technical exclusions.
30	Tison GH, Zhang J, Delling FN, et al. Automated and Interpretable Patient ECG Profiles for Disease Detection, Tracking, and Discovery. Circ Cardiovasc Qual Outcomes. 2019 Sep;12(9):e005289. DOI: 10.1161/circoutcomes.118.005289	Duplicate/companion report	Duplicate/companion of full-text record 26; merge with the primary report before applying clinical criteria.
31	Pan HY, Hsu BWY, Chou CT, et al. Automated Estimation of Computed Tomography-Derived Left Ventricular Mass Using Sex-specific 12-Lead ECG-Based Temporal Convolutional Network. medRxiv [preprint]. 2024. Version 1. DOI: 10.1101/2024.02.19.24303061	Wrong population	No separable athlete cohort or athlete-specific external validation; under the prespecified hierarchy this criterion is applied before target-condition or downstream technical exclusions.
32	Dai H, Hwang HG, Tseng VS. Convolutional neural network based automatic screening tool for cardiovascular diseases using different intervals of ECG signals. Comput Methods Programs Biomed. 2021 May;203:106035. DOI: 10.1016/j.cmpb.2021.106035	Wrong population	No separable athlete cohort or athlete-specific external validation; under the prespecified hierarchy this criterion is applied before target-condition or downstream technical exclusions.
33	Yoon GW, Joo S. Enhanced electrocardiogram classification using Gramian angular field transformation with multi-lead analysis and segmentation techniques. MethodsX. 2025 Apr 8;14:103297. DOI: 10.1016/j.mex.2025.103297	Wrong population	No separable athlete cohort or athlete-specific external validation; under the prespecified hierarchy this criterion is applied before target-condition or downstream technical exclusions.
34	Park J, Kermanshahchi J, Love CJ, et al. Artificial Intelligence for the Detection of Hypertrophic Cardiomyopathy From Standard Electrocardiogram. JACC Adv. 2026 Jun 16;5(7):102914. Online ahead of print. DOI: 10.1016/j.jacadv.2026.102914	Wrong population	No separable athlete cohort or athlete-specific external validation; under the prespecified hierarchy this criterion is applied before target-condition or downstream technical exclusions.
35	Zorzi A, Calore C, Vio R, et al. Accuracy of the ECG for differential diagnosis between hypertrophic cardiomyopathy and athlete's heart: comparison between the European Society of Cardiology (2010) and International (2017) criteria. Br J Sports Med. 2018;52(10):667-673. DOI: 10.1136/bjsports-2016-097438	Wrong index test	Directly addresses differentiation of HCM from athlete's heart, but uses conventional ECG criteria rather than AI/ML applied to ECG.
36	De la Garza-Salazar F, Egenriether B. Clinically interpretable electrovectorcardiographic machine learning criteria for the detection of echocardiographic left ventricular hypertrophy. PLoS One. 2025 Oct 17;20(10):e0334829. DOI: 10.1371/journal.pone.0334829	Wrong population	No separable athlete cohort or athlete-specific external validation; under the prespecified hierarchy this criterion is applied before target-condition or downstream technical exclusions.
37	Khurshid S, Friedman S, Pirruccello JP, et al. Deep Learning to Predict Cardiac Magnetic Resonance-Derived Left Ventricular Mass and Hypertrophy From 12-Lead ECGs. Circ Cardiovasc Imaging. 2021 Jun;14(6):e012281. DOI: 10.1161/circimaging.120.012281	Wrong population	No separable athlete cohort or athlete-specific external validation; under the prespecified hierarchy this criterion is applied before target-condition or downstream technical exclusions.
38	Salatzki J, Schwarz AK, Wolfsteller S, et al. Evaluation of new vectorcardiography algorithms for identifying left ventricular hypertrophy and impaired systolic function. J Electrocardiol. 2026 Jul-Aug;97:154272. DOI: 10.1016/j.jelectrocard.2026.154272	Wrong population	No separable athlete cohort or athlete-specific external validation; under the prespecified hierarchy this criterion is applied before target-condition or downstream technical exclusions.
39	Cai C, Imai T, Hasumi E, et al. One-shot screening: Utilization of a two-dimensional convolutional neural network for automatic detection of left ventricular hypertrophy using electrocardiograms. Comput Methods Programs Biomed. 2024 Apr;247:108097. DOI: 10.1016/j.cmpb.2024.108097	Wrong population	No separable athlete cohort or athlete-specific external validation; under the prespecified hierarchy this criterion is applied before target-condition or downstream technical exclusions.
40	Angelaki E, Kopidakis I, Barmparis GD, et al. Single-lead ECG-based machine learning model for noninvasive detection of left ventricular hypertrophy in hypertension. Hellenic J Cardiol. 2026 Jan 19:S1109-9666(26)00004-7. Online ahead of print. DOI: 10.1016/j.hjc.2026.01.004	Wrong population	No separable athlete cohort or athlete-specific external validation; under the prespecified hierarchy this criterion is applied before target-condition or downstream technical exclusions.
41	Sabovčik F, Cauwenberghs N, Kouznetsov D, et al. Applying machine learning to detect early stages of cardiac remodelling and dysfunction. Eur Heart J Cardiovasc Imaging. 2021 Sep 20;22(10):1208-1217. DOI: 10.1093/ehjci/jeaa135	Wrong population	No separable athlete cohort or athlete-specific external validation; under the prespecified hierarchy this criterion is applied before target-condition or downstream technical exclusions.
42	Kokubo T, Kodera S, Sawano S, et al. Automatic Detection of Left Ventricular Dilatation and Hypertrophy from Electrocardiograms Using Deep Learning. Int Heart J. 2022 Sep 30;63(5):939-947. DOI: 10.1536/ihj.22-132	Wrong population	No separable athlete cohort or athlete-specific external validation; under the prespecified hierarchy this criterion is applied before target-condition or downstream technical exclusions.
43	Pantelidis P, Oikonomou E, Souvaliotis N, et al. Deep learning to diagnose left ventricular hypertrophy from standard, 12-lead ECG signals: a proof-of-concept study. Europace. 2023;25(Suppl 1):euad122.534. Conference abstract. DOI: 10.1093/europace/euad122.534	Wrong population	No separable athlete cohort or athlete-specific external validation; under the prespecified hierarchy this criterion is applied before target-condition or downstream technical exclusions.
44	Angelaki E, Kopidakis I, Barmparis GD, et al. Single-lead ECG-based machine learning model for noninvasive detection of left ventricular hypertrophy in hypertension. Hellenic J Cardiol. 2026 Jan 19:S1109-9666(26)00004-7. Online ahead of print. DOI: 10.1016/j.hjc.2026.01.004	Duplicate/companion report	Duplicate/companion of full-text record 41; merge with the primary report before applying clinical criteria.
45	Nechita LC, Tutunaru D, Nechita A, et al. A Resting ECG Screening Protocol Improved with Artificial Intelligence for the Early Detection of Cardiovascular Risk in Athletes. Diagnostics (Basel). 2025;15(4):477. DOI: 10.3390/diagnostics15040477	Wrong target condition	Athlete population, but the model classifies general cardiovascular risk rather than current HCM.
46	Nechita LC, Tutunaru D, Nechita A, et al. A Resting ECG Screening Protocol Improved with Artificial Intelligence for the Early Detection of Cardiovascular Risk in Athletes. Diagnostics (Basel). 2025;15(4):477. DOI: 10.3390/diagnostics15040477	Duplicate/companion report	Duplicate/companion of full-text record 46; merge with the primary report before applying clinical criteria.
47	Ryu JS, Lee S, Chu Y, et al. CoAt-Mixer: Self-attention deep learning framework for left ventricular hypertrophy using electrocardiography. PLoS One. 2023 Jun 8;18(6):e0286916. DOI: 10.1371/journal.pone.0286916	Wrong population	No separable athlete cohort or athlete-specific external validation; under the prespecified hierarchy this criterion is applied before target-condition or downstream technical exclusions.
48	Naderi H, Kaplan T, van Duijvenboden S, et al. Deep learning to predict left ventricular hypertrophy from the electrocardiogram. Europace. 2026 Feb 3;28(2):euag015. DOI: 10.1093/europace/euag015	Wrong population	No separable athlete cohort or athlete-specific external validation; under the prespecified hierarchy this criterion is applied before target-condition or downstream technical exclusions.
49	Naderi H, Ramírez J, van Duijvenboden S, et al. Diagnostic and prognostic value of ECG-predicted hypertension-mediated left ventricular hypertrophy using machine learning. medRxiv [preprint]. 2024. Version 1. DOI: 10.1101/2024.04.22.24306204	Wrong population	No separable athlete cohort or athlete-specific external validation; under the prespecified hierarchy this criterion is applied before target-condition or downstream technical exclusions.
50	De la Garza-Salazar F, Romero-Ibarguengoitia ME, Rodriguez-Diaz EA, et al. Improvement of electrocardiographic diagnostic accuracy of left ventricular hypertrophy using a Machine Learning approach. PLoS One. 2020 May 13;15(5):e0232657. DOI: 10.1371/journal.pone.0232657	Wrong population	No separable athlete cohort or athlete-specific external validation; under the prespecified hierarchy this criterion is applied before target-condition or downstream technical exclusions.
51	De la Garza Salazar F, Romero Ibarguengoitia ME, Azpiri López JR, et al. Optimizing ECG to detect echocardiographic left ventricular hypertrophy with computer-based ECG data and machine learning. PLoS One. 2021 Nov 30;16(11):e0260661. DOI: 10.1371/journal.pone.0260661	Wrong population	No separable athlete cohort or athlete-specific external validation; under the prespecified hierarchy this criterion is applied before target-condition or downstream technical exclusions.
52	Ning H, Yu Q, Kong F, et al. Artificial intelligence electrocardiogram as a novel screening tool to detect and longitudinally monitor high-risk echocardiographic features in hypertrophic cardiomyopathy. Quant Imaging Med Surg. 2025 Apr 1;15(4):2905-2916. DOI: 10.21037/qims-24-1638	Wrong population	No separable athlete cohort or athlete-specific external validation; under the prespecified hierarchy this criterion is applied before target-condition or downstream technical exclusions.
53	Kaiser W, Faber TS, Findeis M. Automatic learning of rules. A practical example of using artificial intelligence to improve computer-based detection of myocardial infarction and left ventricular hypertrophy in the 12-lead ECG. J Electrocardiol. 1996;29 Suppl:17-20. DOI: 10.1016/s0022-0736(96)80004-5	Wrong population	No separable athlete cohort or athlete-specific external validation; under the prespecified hierarchy this criterion is applied before target-condition or downstream technical exclusions.
54	Angelaki E, Marketou M, Barmparis G, et al. Detection of left ventricular hypertrophy on the ECG through machine learning with a focus on obesity. Eur Heart J Digit Health. 2022;3(4):ztac076.2772. Conference abstract reprint. DOI: 10.1093/ehjdh/ztac076.2772	Wrong population	No separable athlete cohort or athlete-specific external validation; under the prespecified hierarchy this criterion is applied before target-condition or downstream technical exclusions.
55	Naderi H, Ramírez J, van Duijvenboden S, et al. Diagnostic and prognostic value of ECG-predicted hypertension-mediated left ventricular hypertrophy using machine learning. medRxiv [preprint]. 2024. Version 1. DOI: 10.1101/2024.04.22.24306204	Duplicate/companion report	Duplicate/companion of full-text record 50; merge with the primary report before applying clinical criteria.
56	Smaranda AM, Drăgoiu TS, Caramoci A, et al. Artificial Intelligence in Sports Medicine: Reshaping Electrocardiogram Analysis for Athlete Safety - A Narrative Review. Sports (Basel). 2024 May 26;12(6):144. DOI: 10.3390/sports12060144	Wrong publication type/no original data	Narrative review without original participant-level diagnostic-accuracy data.

Appendix C: Ongoing directly relevant study

ClinicalTrials.gov identifier: NCT06290570

Status/design: Prospective study; no result-bearing report was available at the evidence lock.

Population/target condition: HCM versus physiological athlete's heart.

Index test: Standard 12-lead ECG plus consumer-facing ECG devices, including Apple Watch and KardiaMobile, evaluated using AI-ECG methods.

Planned outcomes: Diagnostic-performance measures, including true-positive, true-negative, false-positive, and false-negative classifications.

Contribution to the present review: No diagnostic-performance results were available; therefore, the study did not contribute data to the qualitative or quantitative evidence synthesis.

Appendix D: Final consensus QUADAS-2 assessment

The included Fatunde et al. report underwent QUADAS-2 assessment. The final consensus judgments were as follows:

Patient selection: Risk of bias: Unclear; Applicability concern: Low. Consecutive or random enrollment and exclusions were not sufficiently reported. However, the athlete-screening population was directly relevant to the review question.

Index test: Risk of bias: Unclear; Applicability concern: Low. The AI-ECG index test matched the review question, but model version, locking status, and a prespecified HCM positivity threshold were not reported.

Reference standard: Risk of bias: Unclear; Applicability concern: Unclear. Echocardiography was acceptable in principle, but HCM diagnostic criteria, reader blinding, and ECG-to-imaging timing were not reported. Applicability was adjudicated as unclear.

Flow and timing: Risk of bias: High; Applicability concern: Not applicable. Only 99 of 845 athletes underwent the reported echocardiographic reference standard, creating substantial partial-verification concern.

Overall: Risk of bias: High; Applicability concern: Unclear. Overall high risk of bias was driven primarily by partial verification. Overall applicability was considered unclear because reporting of the reference standard was insufficient. Final consensus followed adjudication where required.

Appendix E: Information sources and original June 2026 retrieval counts

PubMed/MEDLINE: June 22, 2026: 323 records. The June archive is authoritative. The exact executed historical syntax was not fully recoverable; broader retrieval was documented.

Embase.com: June 19, 2026: 83 records, including one within-source duplicate. The executed syntax was partly reconstructed from the archived search documentation.

CENTRAL: June 22, 2026: 119 records. The historical Search Manager sequence was recovered; the June archive remained authoritative.

Scopus: June 19, 2026: 127 records. Executed syntax was reconstructed from the archive/search log.

Web of Science Core Collection, Science Citation Index Expanded: June 19, 2026: 48 records. Executed syntax was reconstructed from the archive/search log.

Europe PMC: June 22, 2026: 183 records. Search syntax was documented.

IEEE Xplore: June 22, 2026: 79 records. Search syntax was reconstructed from the archive/search log.

ClinicalTrials.gov Query A: June 22, 2026: 12 records. Focused HCM + AI registry query.

ClinicalTrials.gov Query B: June 22, 2026: 26 records. HCM/LVH + ECG + AI registry query.

ClinicalTrials.gov Query C: June 22, 2026: 22 records. Athlete + ECG + AI registry query. The three ClinicalTrials.gov searches yielded 60 raw occurrences and 49 unique registrations before cross-source deduplication.

WHO ICTRP: June 22, 2026: 2 records. Both records were also present in ClinicalTrials.gov.

These original June counts were retained for the formal review and PRISMA flow.

Appendix F: Search strategies and documentation

The formal review used the original June 2026 database and registry exports. Search dates and raw retrieval counts are retained below and in Appendix E. The accompanying documentation archive contains the original June exports, the archived reporting log, the recovered CENTRAL transcription, the operational search plan, and the available Google Scholar result snapshots.

The archived reporting log contains reconstructed strategies and is not a complete original platform history. Queries are therefore labelled according to their provenance. Recovered CENTRAL syntax is distinguished from reconstructed queries and from later source-specific reproducibility audits. The latter were not incorporated as an all-source formal search update and do not alter the June PRISMA counts.

The operational plan specified no date or language restrictions and covered electrocardiography, AI/ML, HCM and related hypertrophic phenotypes, and athletes or sports screening. The archived retrieval was broader than a mandatory four-concept intersection in some sources. Where an original platform history was unavailable, an absence of additional filters could not be independently verified from a bibliographic export alone. CENTRAL/Trials and the Web of Science SCIE index are identified separately below.

PubMed

Original search date: 22 June 2026. Original exported records: 323.

Strategy provenance: Reconstructed archive-consistent reporting strategy. The exact query executed in June was not fully recoverable. The strategy below was used for the 17 August record-recovery audit; it is not represented as a preserved June platform history.

Reported Strategy

("Electrocardiography"[Mesh] OR electrocardiograph*[Title/Abstract] OR electrocardiogram*[Title/Abstract] OR electrocardiographic*[Title/Abstract] OR ECG[Title/Abstract] OR EKG[Title/Abstract] OR "12-lead ECG"[Title/Abstract] OR "12 lead ECG"[Title/Abstract] OR "single-lead ECG"[Title/Abstract] OR "single lead ECG"[Title/Abstract] OR "wearable ECG"[Title/Abstract] OR "smartwatch ECG"[Title/Abstract] OR "ambulatory ECG"[Title/Abstract] OR Holter[Title/Abstract] ) AND ( "Artificial Intelligence"[Mesh] OR "Machine Learning"[Mesh] OR "Deep Learning"[Mesh] OR "Neural Networks, Computer"[Mesh] OR "artificial intelligence"[Title/Abstract] OR AI[Title/Abstract] OR "AI-enabled"[Title/Abstract] OR "AI-enhanced"[Title/Abstract] OR "AI-based"[Title/Abstract] OR "machine learning"[Title/Abstract] OR "deep learning"[Title/Abstract] OR "neural network*"[Title/Abstract] OR "convolutional neural network*"[Title/Abstract] OR CNN[Title/Abstract] OR algorithm*[Title/Abstract] OR classifier*[Title/Abstract] OR "automated classifier"[Title/Abstract] ) AND ( "Cardiomyopathy, Hypertrophic"[Mesh] OR "hypertrophic cardiomyopath*"[Title/Abstract] OR "hypertrophic obstructive cardiomyopath*"[Title/Abstract] OR HCM[Title/Abstract] OR HOCM[Title/Abstract] OR "idiopathic hypertrophic subaortic stenosis"[Title/Abstract] OR "athlete's heart"[Title/Abstract] OR "athletes' heart"[Title/Abstract] OR "athletic heart"[Title/Abstract] OR "left ventricular hypertroph*"[Title/Abstract] OR LVH[Title/Abstract])

Embase

Original search date: 19 June 2026. Original exported records: 83.

Strategy provenance: Partly reconstructed strategy informed by the archived documentation and original export. A completed record-level recovery check was not documented. The file labelled as a 17 August export was byte-for-byte identical to the June export and does not establish a later search execution.

Reported Strategy

( 'electrocardiography'/exp OR electrocardiograph*:ti,ab,kw OR electrocardiogram*:ti,ab,kw OR electrocardiographic*:ti,ab,kw OR ecg:ti,ab,kw OR ekg:ti,ab,kw OR '12-lead ecg':ti,ab,kw OR '12 lead ecg':ti,ab,kw OR 'single-lead ecg':ti,ab,kw OR 'wearable ecg':ti,ab,kw OR 'smartwatch ecg':ti,ab,kw OR 'ambulatory ecg':ti,ab,kw OR holter:ti,ab,kw ) AND ( 'artificial intelligence'/exp OR 'machine learning'/exp OR 'deep learning':ti,ab,kw OR 'neural network*':ti,ab,kw OR 'convolutional neural network*':ti,ab,kw OR cnn:ti,ab,kw OR algorithm*:ti,ab,kw OR classifier*:ti,ab,kw OR 'automated classifier':ti,ab,kw OR 'ai-enabled':ti,ab,kw OR 'ai-enhanced':ti,ab,kw OR 'ai-based':ti,ab,kw ) AND ( 'hypertrophic cardiomyopathy'/exp OR 'hypertrophic cardiomyopath*':ti,ab,kw OR 'hypertrophic obstructive cardiomyopath*':ti,ab,kw OR hcm:ti,ab,kw OR hocm:ti,ab,kw OR 'idiopathic hypertrophic subaortic stenosis':ti,ab,kw OR 'athlete heart':ti,ab,kw OR 'athlete''s heart':ti,ab,kw OR 'athletes'' heart':ti,ab,kw OR 'athletic heart':ti,ab,kw OR 'left ventricular hypertroph*':ti,ab,kw OR lvh:ti,ab,kw )

CENTRAL

Original search date: 22 June 2026. Original exported records: 119.

Strategy provenance: Complete recovered Search Manager transcription, derived from the historical screenshots and checked against the final audit workbook. This sequence supersedes the earlier reporting-log reconstruction that identified the final line as #22.

Product: Cochrane Library, CENTRAL/Trials. No date or language restriction is recorded in the recovered audit documentation. The sequence below reproduces all sets required for the final union, retaining the recovered numbering #4 through #25. The displayed counts belong to the recovered Search Manager history; the original June RIS export contained 119 records and remains the formal PRISMA source count.

#4   Displayed count: 42,973

(electrocardiograph* OR electrocardiogram* OR electrocardiographic* OR ECG OR EKG OR "12-lead ECG" OR "12 lead ECG" OR "single-lead ECG" OR "single lead ECG" OR "wearable ECG" OR "smartwatch ECG" OR "ambulatory ECG" OR Holter):ti,ab,kw

#5   Displayed count: 30,552

("artificial intelligence" OR "machine learning" OR "deep learning" OR (neural NEXT network*) OR (convolutional NEXT neural NEXT network*) OR (deep NEXT neural NEXT network*) OR CNN OR algorithm* OR classifier* OR "automated classifier" OR "automated detection" OR "computer-aided diagnosis" OR "AI-enabled" OR "AI-enhanced" OR "AI-based"):ti,ab,kw

#6   Displayed count: 2,779

((hypertrophic NEXT cardiomyopath*) OR (hypertrophic NEXT obstructive NEXT cardiomyopath*) OR HCM OR HOCM OR "idiopathic hypertrophic subaortic stenosis" OR "athlete's heart" OR "athletes' heart" OR "athletic heart" OR (left NEXT ventricular NEXT hypertroph*) OR LVH):ti,ab,kw

#7   Displayed count: 192,053

(athlete* OR athletic* OR sport* OR exercis* OR "sports cardiology" OR "preparticipation screening" OR "pre-participation screening" OR "cardiovascular screening" OR "preparticipation cardiovascular screening"):ti,ab,kw

#8   Displayed count: 16

#4 AND #5 AND #6

#9   Displayed count: 103

#4 AND #5 AND #7

#10   Displayed count: 117

#8 OR #9

#11   Displayed count: 10,714

MeSH descriptor: (Electrocardiography) explode all trees

#12   Displayed count: 4,318

MeSH descriptor: (AI) explode all trees

#13   Displayed count: 1,434

MeSH descriptor: (ML) explode all trees

#14   Displayed count: 387

MeSH descriptor: (Cardiomyopathy, Hypertrophic) explode all trees

#15   Displayed count: 1,131

MeSH descriptor: (Hypertrophy, Left Ventricular) explode all trees

#16   Displayed count: 2,145

MeSH descriptor: (Athletes) in all MeSH products

#17   Displayed count: 24,242

MeSH descriptor: (Sports) explode all trees

#18   Displayed count: 44,224

MeSH descriptor: (Exercise) explode all trees

#19   Displayed count: 43,084

#4 OR #11

#20   Displayed count: 32,050

#5 OR #12 OR #13

#21   Displayed count: 3,217

#6 OR #14 OR #15

#22   Displayed count: 197,680

#7 OR #16 OR #17 OR #18

#23   Displayed count: 17

#19 AND #20 AND #21

#24   Displayed count: 105

#19 AND #20 AND #22

#25   Displayed count: 120

#23 OR #24

Final architecture: (ECG AND AI/ML AND HCM/LVH) OR (ECG AND AI/ML AND athlete/sports), combining free text with MeSH. The displayed final Search Manager count of 120 is reported separately from the 119 records in the original June export. The August export and record-recovery comparison are described below.

Scopus

Original search date: 19 June 2026. Original exported records: 127.

Strategy provenance: Reconstructed TITLE-ABS-KEY strategy from the archived reporting log and export. Exact historical syntax and record-level recovery were not fully verified.

Reported Strategy

TITLE-ABS-KEY(( electrocardiography OR electrocardiogram OR electrocardiographic OR ECG OR EKG OR "12-lead ECG" OR "12 lead ECG" OR "single-lead ECG" OR "wearable ECG" ) AND ( "artificial intelligence" OR "machine learning" OR "deep learning" OR "neural network" OR "artificial neural network" OR "convolutional neural network" OR "deep neural network" OR "AI-ECG" OR "AI ECG" OR "automated classification" OR "automated detection" ) AND ( "hypertrophic cardiomyopathy" OR "hypertrophic obstructive cardiomyopathy" OR HCM OR HOCM OR "left ventricular hypertrophy" OR LVH OR "athlete's heart" OR "athletic heart" ))

Web of Science SCIE

Original search date: 19 June 2026. Original exported records: 48.

Strategy provenance: Reconstructed topic-search strategy from the archived reporting log and export. The documented index was Science Citation Index Expanded. Exact historical syntax and record-level recovery were not fully verified.

Index: Science Citation Index Expanded. No other restrictions were specified in the available reporting log.

Reported Strategy

TS=((electrocardiography OR electrocardiogram OR electrocardiographic OR ECG OR EKG OR "12-lead ECG" OR "12 lead ECG" OR "single-lead ECG" OR "wearable ECG") AND ("artificial intelligence" OR "machine learning" OR "deep learning" OR "neural network" OR "artificial neural network" OR "convolutional neural network" OR "deep neural network" OR "AI-ECG" OR "AI ECG" OR "automated classification" OR "automated detection") AND ("hypertrophic cardiomyopathy" OR "hypertrophic obstructive cardiomyopathy" OR HCM OR HOCM OR "left ventricular hypertrophy" OR LVH OR "athlete's heart" OR "athletic heart"))

Europe PMC

Original search date: 22 June 2026. Original exported records: 183.

Strategy provenance: Strategy documented in the archived reporting log. A separate original platform history and a complete August record-recovery rerun were not available in the reviewed documentation.

Reported strategy:

TITLE_ABS:((electrocardiography OR electrocardiogram OR electrocardiographic OR ECG OR EKG OR "12-lead ECG" OR "12 lead ECG" OR "single-lead ECG" OR "wearable ECG") AND ("artificial intelligence" OR "machine learning" OR "deep learning" OR "neural network" OR "artificial neural network" OR "convolutional neural network" OR "deep neural network" OR "AI-ECG" OR "AI ECG" OR "automated classification" OR "automated detection") AND ("hypertrophic cardiomyopathy" OR "hypertrophic obstructive cardiomyopathy" OR HCM OR HOCM OR "left ventricular hypertrophy" OR LVH OR "athlete's heart" OR "athletic heart"))

IEEE Xplore

Original search date: 22 June 2026. Original exported records: 79.

Strategy provenance: Reconstructed All Metadata strategy from the archived reporting log and export. Exact historical syntax and record-level recovery were not fully verified.

Reported Strategy

"All Metadata":((electrocardiography OR electrocardiogram OR electrocardiographic OR ECG OR EKG OR "12-lead ECG" OR "12 lead ECG" OR "single-lead ECG" OR "wearable ECG") AND ("artificial intelligence" OR "machine learning" OR "deep learning" OR "neural network" OR "artificial neural network" OR "convolutional neural network" OR "deep neural network" OR "AI-ECG" OR "AI ECG" OR "automated classification" OR "automated detection") AND ("hypertrophic cardiomyopathy" OR "hypertrophic obstructive cardiomyopathy" OR HCM OR HOCM OR "left ventricular hypertrophy" OR LVH OR "athlete's heart" OR "athletic heart"))

ClinicalTrials.gov Query A

Original search date: 22 June 2026. Original exported records: 12.

Strategy provenance: Reconstructed focused registry query. The original export is available, but the exact platform query was not embedded in that export and a complete original platform history was not recovered.

Reported Strategy

("hypertrophic cardiomyopathy" OR HCM) AND ("artificial intelligence" OR "machine learning" OR "deep learning")

ClinicalTrials.gov Query B

Original search date: 22 June 2026. Original exported records: 26.

Strategy provenance: Reconstructed HCM/LVH, ECG, and AI registry query. The original export is available, but the exact platform query was not embedded in that export and a complete original platform history was not recovered.

Reported Strategy

("hypertrophic cardiomyopathy" OR HCM OR "left ventricular hypertrophy" OR LVH) AND (electrocardiogram OR ECG) AND ("artificial intelligence" OR "machine learning" OR "deep learning")

ClinicalTrials.gov Query C

Original search date: 22 June 2026. Original exported records: 22.

Strategy provenance: Reconstructed athlete, ECG, and AI registry query. The original export is available, but the exact platform query was not embedded in that export and a complete original platform history was not recovered.

Reported Strategy

(athlete OR athletes OR sport OR "athlete's heart" OR "athletic heart") AND (electrocardiogram OR ECG) AND ("artificial intelligence" OR "machine learning" OR "deep learning")

WHO ICTRP

Original search date: 22 June 2026. Original exported records: 2.

Strategy provenance: Combined query reconstructed for reporting. The original two-record CSV is available, but the exact executed platform sequence was not recovered. The operational plan listed six short WHO ICTRP queries; these planned queries are preserved in the supporting search-plan PDF and are not treated as a recovered executed history.

Reported Strategy

(ECG OR electrocardiogram) AND ("artificial intelligence" OR "machine learning" OR "deep learning") AND ("hypertrophic cardiomyopathy" OR HCM OR "left ventricular hypertrophy" OR LVH OR "athlete's heart")

Assessment of Correspondence With Archived Retrieval

Correspondence was assessed through comparisons with the archived June records rather than through matching total result counts alone. The available documentation records identifier-level recovery for PubMed and identifier/title comparisons for CENTRAL. No completed equivalent recovery check is documented for every other source.

PubMed: An initial reconstructed query retrieved 179 records and recovered 174 of the 323 original June PMIDs, leaving 149 unrecovered; it was not accepted as equivalent to the original retrieval. An archive-consistent three-block strategy was then tested on 17 August 2026. It retrieved 331 records and recovered all 323 original June PMIDs, with zero original PMIDs missing and eight additional records. All eight additional records were screened, and none of them met the primary review eligibility criteria. The successful recovery supports correspondence with the archived record set but does not prove identity with the exact query executed in June.

CENTRAL: The 17 August 2026 export contained 120 records and recovered all 119 original June records, with zero original records missing and one additional record. The additional record, CN-03026575 / ISRCTN11121053, concerned triiodothyronine in heart failure and was excluded for wrong population. These findings support recovery of the original CENTRAL record set without replacing the original June count of 119.

Embase: The file labelled Group2_Embase_2026-08-17.ris was identical to the original Group2_Embase_2026-06-19.ris in size, SHA-256 checksum, and record content. It therefore did not constitute a new rerun or an independent correspondence test. The original 83-record export remains authoritative.

Scopus, Web of Science, IEEE Xplore, and the trial registries: The exact original platform histories were not fully recovered, and no complete identifier-level August recovery check was documented. Europe PMC syntax was documented in the archived reporting log, but a complete independent rerun was not available. Reconstruction gaps remain a limitation of reproducibility.

The PubMed and CENTRAL checks were source-specific reproducibility audits. They were not an all-source search update, and their additional records were not added to the June PRISMA flow. A broader reconstructed query, a similar result count, or recovery of an archived record set does not establish complete historical search reproduction or proven search sensitivity.

Available Supporting Search Documentation

Original June source exports are supplied unchanged. The archived Group2_Search_Log.xlsx is supplied as an historical reporting log; its reconstructed queries retain their original labels. Its earlier CENTRAL statement about a final line #22 is superseded by the recovered #25 = #23 OR #24 transcription. The available source archive does not contain separately preserved original platform histories for every database or the CENTRAL screenshot image files; the recovered transcription and its provenance are supplied without presenting them as original image files.

The operational search-plan PDF is provided as planned methodology, distinct from execution evidence. The available Google Scholar result snapshots are also supplied. They contain 290 displayed result entries across 29 saved pages; these displayed entries are not automatically unique additional records and do not replace the 18 supplementary records documented in the review screening set.

Appendix G: ECG definitions and positivity thresholds

This table summarizes the HCM target or reference definitions, conventional ECG interpretation criteria, and AI positivity thresholds used in the athlete and clinical reports discussed in this review [4-7,16].

**Table 3:** Electrocardiogram definitions and positivity thresholds in athlete and clinical hypertrophic cardiomyopathy reports. Reports [4-7] provide conventional ECG or indirect clinical context; Fatunde et al. [16] is the only published report included in the primary qualitative synthesis. Imaging diagnostic criteria differ from ECG positivity thresholds. AI thresholds are model-specific and cannot be assumed transferable to athletes. Cross-study results do not establish superiority over conventional athlete ECG criteria. ACC/AHA: American College of Cardiology/American Heart Association; AI: artificial intelligence; AI-ECG: artificial intelligence-enhanced electrocardiography; AUC: area under the receiver operating characteristic curve; CMR: cardiac magnetic resonance imaging; ECG: electrocardiogram; ESC: European Society of Cardiology; HCM: hypertrophic cardiomyopathy; PPV: positive predictive value; QRS: ventricular depolarization complex.

Report and population	HCM target or reference definition	ECG interpretation rule or AI positivity threshold	Reported diagnostic evidence
Zorzi et al. [4]. Conventional ECG comparison: 200 HCM patients and 563 athletes.	HCM versus physiological athlete's heart; comparison of conventional ECG interpretation criteria.	2010 ESC versus 2017 International criteria. Isolated high QRS voltage was non-pathological under both. Isolated left atrial enlargement or left axis deviation, and Q waves ≥4 mm but <25% of the R wave and <0.04 s, were non-pathological under the International criteria.	2010 ESC: sensitivity 95.5%, specificity 86.9%. 2017 International: sensitivity 93%, specificity 95.9%. These are categorical ECG rules, not AI probability thresholds.
Siontis et al. [5]. International clinical validation: 4,640 patients (773 HCM and 3,867 controls).	Cardiologist-adjudicated HCM supported by echocardiography or CMR: wall thickness ≥15 mm without another cause of hypertrophy, or ≥13 mm with a family history of HCM or a pathogenic HCM mutation.	AI probability >11% (0.11), carried forward from model derivation. A secondary 18% (0.18) cutoff was optimized in the combined validation cohort using the Youden index.	AUC 0.922. At 11%: sensitivity 82.8%, specificity 87.7%. At the secondary 18% cutoff: sensitivity 80.6%, specificity 90.9%.
Lewontin et al. [6]. HCM referral center: 404 patients (230 HCM and 174 controls); 3,499 ECGs.	ACC/AHA guideline-based HCM diagnosis, confirmed by manual chart review. Numerical wall-thickness criteria were not restated in the report.	Probability-based AI classification. The numerical positivity cutoff was not reported; the authors state that cutoffs were designed to favor specificity and PPV.	AUC 0.91 using either the mean probability across each patient's ECGs or the earliest ECG. Reported sensitivity 67% and specificity 95%.
Park et al. [7]. Clinical CMR cohort: 150 HCM patients and 83 controls.	Physician-reviewed records and CMR: maximal wall thickness ≥15 mm, or ≥13 mm with a family history or genetic HCM diagnosis, without a hemodynamic explanation for the hypertrophy.	Prespecified AI probability cutoff of 0.84, chosen to favor high specificity.	AUC 0.946; sensitivity 58%, specificity 100% at the prespecified operating point.
Fatunde et al. [16]. Pre-participation screening: 845 collegiate or professional athletes.	Follow-up echocardiography in 99 athletes. HCM imaging diagnostic criteria were not reported.	HCM positivity threshold not reported. An athlete-specific AI threshold was not established by this report.	HCM-specific sensitivity, specificity, AUC, and participant-level 2 × 2 diagnostic data were not reported. Included in qualitative synthesis only.
